# Anti-CD19 CAR T-cell therapy for autoimmune diseases: from exploratory clinical trials to quantitative pharmacodynamic modeling development

**DOI:** 10.3389/fimmu.2026.1823722

**Published:** 2026-09-15

**Authors:** Yuqin Sun, Xiao Heng, Guang Hu, Xiumei Bo

**Affiliations:** 1 The First Clinical College, Xuzhou Medical University, Xuzhou, China; 2 Nanjing IASO Biotherapeutics Co Ltd, Nanjing, China; 3 National Demonstration Center for Experimental Basic Medical Science Education, Xuzhou Medical University, Xuzhou, China

**Keywords:** Anti-CD19 CAR T, autoimmune diseases, immunotherapy, individualized treatment, quantitative systems pharmacology models

## Abstract

Anti-CD19 CAR T-cell therapy has produced major advances in treating hematologic malignancies. Recently, researchers have explored its use in autoimmune diseases. However, the complex pathogenesis and high heterogeneity of these disorders create substantial uncertainty about treatment efficacy and safety in clinical settings. Quantitative systems pharmacology (QSP) models integrate multiple pathophysiological mechanisms and map real patients onto virtual “digital twins” to simulate the *in vivo* behavior of a given CAR T-cell dose after infusion and to predict benefits and risks. Such models can also inform optimization of infusion dosing, advancing personalized therapy. This review summarizes current applications of anti-CD19 CAR T cells in autoimmune diseases and surveys the state of quantitative mathematical modeling in the field. Building on existing models, we propose an idealized QSP framework intended to guide the development of more refined, actionable models.

## 1 Introduction

Autoimmune diseases are heterogeneous, mechanistically complex and lack targeted therapies (1). Although recent years have seen promising advances in therapies, clinical treatment still faces two challenges: the severe side effects associated with broad-spectrum immunosuppressants such as cyclophosphamide, and the high cost and uncertain long-term risks of newer biologics, including monoclonal antibodies and antibody–drug conjugates (2, 3).

Building on recent breakthroughs in hematologic malignancies, cell therapy has recently opened new avenues for treating autoimmune diseases (4). In particular, chimeric antigen receptor T-cell (CAR T) technology has open the possibility of long-term remissions in autoimmune disease (5). A flowchart illustrating the application of CAR T-cell therapy in autoimmune diseases is shown in **Figure 1**.

**Figure 1:**
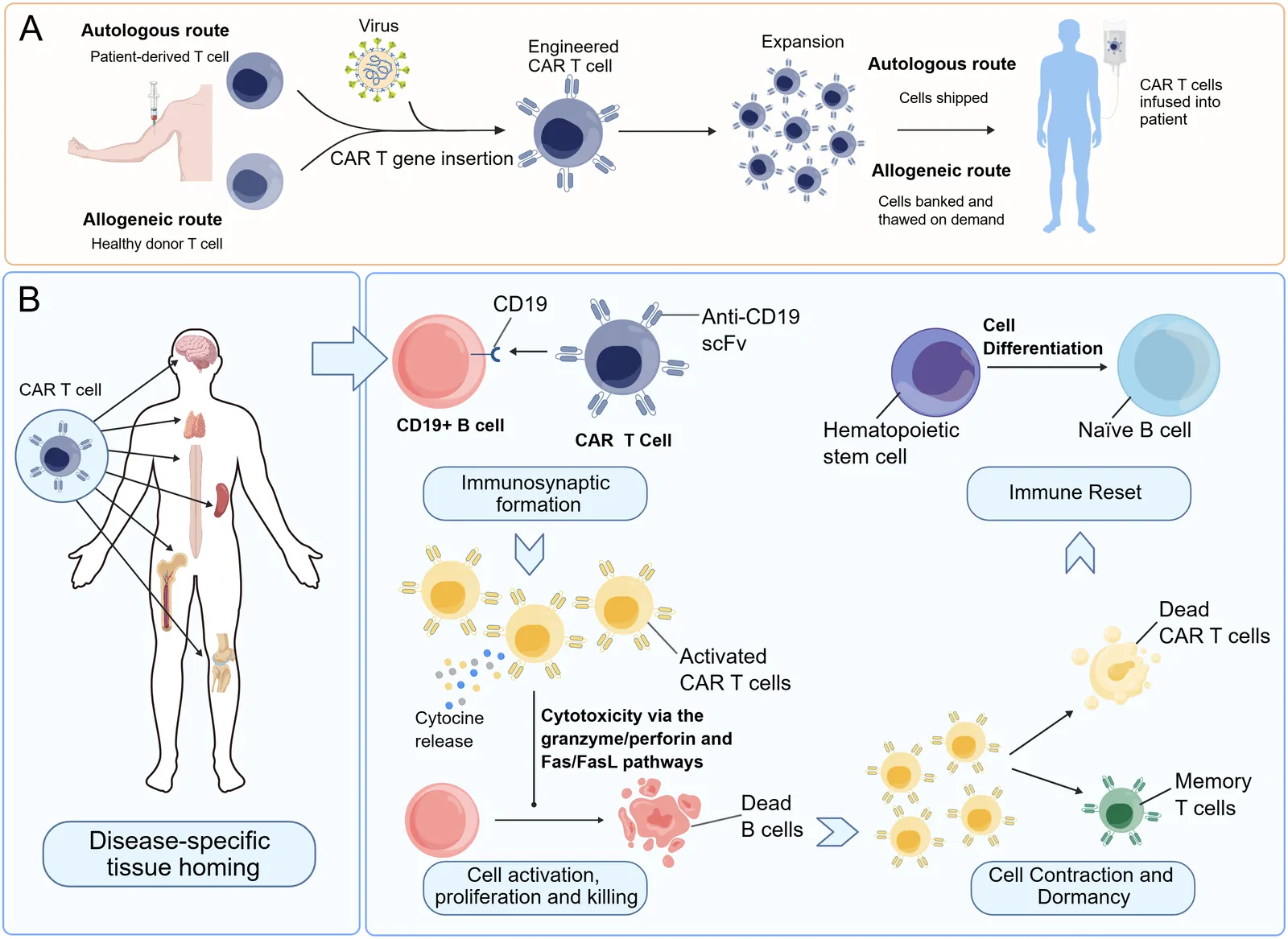
Schematic diagram of the manufacturing and mechanism of chimeric antigen receptor (CAR) T-cell therapy. **(A)** *In vitro* preparation process for anti-CD19 CAR T-cell therapy, showing autologous and allogeneic approaches. First, T cells are collected from the patient or from a healthy donor. The anti-CD19 CAR transgene is introduced by a viral vector, and the modified T cells are expanded *in vitro*. The cells are then cryopreserved or shipped from a cell bank, and finally reinfused into the patient. **(B)** *In vivo* mechanisms of anti-CD19 CAR T-cell therapy in autoimmune diseases. After intravenous reinfusion, CAR T cells first home to lymphoid organs and to disease-specific target tissues, such as synovial membranes and kidneys. At these sites, the anti-CD19 scFv on CAR T cells recognizes CD19+ B cells and forms an immunological synapse. This interaction activates the CAR T cells and drives robust proliferation, leading to target-cell elimination via the granzyme–perforin and Fas/FasL pathways. Activation and cytotoxicity are accompanied by release of large amounts of cytokines, including IL-6, IFN-γ, and TNF-α, which can produce the risk of CRS and ICANS. If normal tissues express low levels of CD19 or if cross-reactivity occurs during targeting, off-target damage to healthy tissues may result. Following B-cell depletion, hematopoietic stem cells regenerate a pool of naïve, unsensitized B cells and the immune system is reset. Most CAR T cells then decline in number from lack of antigenic stimulation, while a small fraction differentiates into memory T cells that surveil for recurrence. If memory T cells are excessive or persist abnormally, they can perturb maturation of newly formed B cells and increase the risk of infection and hypogammaglobulinemia.

CAR T-cell therapy represented a first-class breakthrough in the treatment of B-cell leukemia (6). In autoimmune diseases, the elimination of B cells stops autoantibody production at its source, thereby playing a key role in disease management (7). Furthermore, CAR T therapy can provide disease remission without recurrence by targeting memory B cells (8). This result motivated researchers to investigate CAR T therapy for the treatment of the autoimmune disease (9). Clinical studies show that CAR T-cell therapy can provide drug-free long-term remission for refractory autoimmune disease treatment (5, 10, 11). To date, 197 CAR T cell clinical trials targeting autoimmune diseases have been registered on ClinicalTrials.gov. (searched using ‘CAR T’ and ‘autoimmune disease’; https://clinicaltrials.gov/search?cond=autoimmunedisease&term=CART&viewType=Card). Among these, most clinical trials selected anti-CD19 CAR T-cell products and achieved favorable efficacy (12).

CAR T-cell therapy exhibits distinct pharmacological behavior—marked by *in vivo* expansion, contraction, and long-term persistence—and patient-specific factors strongly influence expansion magnitude and the risk of adverse events such as cytokine release syndrome (13, 14). Consequently, dosing strategies for CAR T must differ from those used for conventional small molecules and biologics (15). There is an urgent need to move from fixed-dose regimens to personalized dosing schemes. Individualized dosing can tailor administration to each patient’s condition to improve efficacy and can substantially reduce the likelihood of severe toxicities such as cytokine storms (16, 17). Clinically, however, personalized CAR T administration remains constrained by unclear dose–exposure–response relationships and unpredictable cell dynamics, which significantly hinder its routine individualized application (18, 19).

In recent years, Quantitative systems pharmacology models have addressed these issues by integrating short-term clinical data with externally validated cohorts to characterize cellular dynamics between immunophenotypic subgroups and provide dose guidance for clinical use (20). This results in CAR T development has moved away from trial-and-error methods based on clinical experience and now support dose optimization, response prediction, and individualized treatment regimens for future trials (21).

## 2 Clinical exploration of anti-CD19 CAR T-cell therapy in autoimmune diseases

### 2.1 Mechanism of action of anti-CD19 CAR T-cell therapy in autoimmune diseases

CD19 regulates B cell receptor signaling and hence B cell proliferation (22, 23). Since CD19 is expressed continuously at all stages of B cell development, it is the most popular target for CAR T-cell therapy (24, 25).

In autoimmune diseases, CD19 expression patterns are quite similar on normal and abnormally activated B cells, while CD19 density is much higher on patients B cells than in healthy individuals (26). This provides biological reason for adapting targeting strategies developed in oncology to autoimmune disease and allows a wider safety window for CAR T therapy (5).

Research demonstrates that anti-CD19 CAR T exhibits a unique biphasic “clear-rebuild” action pattern in autoimmune diseases (27–29). In the initial phase, CD19+ B cells—including memory B cells producing autoantibodies and plasma blasts—are rapidly eliminated via Fas/FasL signaling and granzyme B-mediated cytotoxicity (30). Concurrent with B-cell depletion, serum levels of specific autoantibodies (e.g., anti-Jo-1 antibodies) significantly decrease, while immunoglobulin subclasses (IgA, IgG, IgM) partially recover. Subsequently, the immune regulation phase commences. CAR T cells secrete anti-inflammatory factors like IL-10 and TGF-β to induce the regeneration of regulatory B cells (Bregs) while promoting the expansion of regulatory T cells (Tregs), thereby reestablishing immune tolerance (31, 32).

The clinical results show that anti-CD19 CAR T induces massive B-cell depletion, significantly reduces immune complex deposition and ameliorates target organ injuries (e.g. renal inflammation) (33). In patients with SLE patients, the complete B-cell depletion was observed after treatment in peripheral blood and lymph nodes biopsies.

### 2.2 Clinical data summary of anti-CD19 CAR T in autoimmune diseases along with its pharmacokinetic and pharmacodynamic evaluation

In order to assess current clinical effectiveness of anti-CD19 CAR T treatment of autoimmune diseases and guide future quantitative model modeling, we retrospectively reviewed 42 real-world cases reported in literature. We evaluated Pharmacokinetics and Pharmacodynamics of CAR T cells and characterized immune reconstitution after injection in **Table 1** (34–46).

**Table 1:** Clinical data summary of Anti-CD19 CAR T therapy in multiple autoimmune diseases.

References	Diseases	Cell Count	Cmax (cells/μL)	T1/2 (d)	AUC (0-t)	Tmax(d)
(34)	SLE	25×10 ^6^	19	7.44	154	8
(34)	SLE	25×10 ^6^	228	3.32	2376	29
(34)	SLE	25×10 ^6^	10	9	156	11
(34)	SLE	75×10 ^6^	8	6.59	97	8
(34)	SLE	75×10 ^6^	88	7.98	471	11.5
(34)	SLE	75×10 ^6^	28	9.28	291	8
(34)	SLE	50×10 ^6^	13	3.81	101	11.5
(34)	SLE	100×10 ^6^	17	8.35	181	8
(35)	SLE	1×10^6^/kg	75	41.6	3186	22
(35)	SLE	1×10^6^/kg	16	22.18	1152	60
(35)	SLE	1×10 ^6^/kg	264	35.93	2711	14.5
(35)	SLE	1×10 ^6^/kg	71	78.43	1722	10.5
(36)	MS	——	383	26.35	5503	7
(36)	MS	——	58	17.06	669	16
(37)	LEMS	1.2×10 ^8^	192	3.69	1661	7
(38)	MG	1×10 ^8^	251	3.68	1498	9
(38)	MG	1×10 ^8^	579	3.42	1724	8
(39)	SLE	——	117	2.52	868	8
(40)	ASS	1.23×10 ^6^/kg	105	17.39	800	7
(41)	SPS	1×10 ^8^	171	0.84	656	14
(42)	MG	1×10 ^8^	131	16.82	1142	16
(43)	JDM	1×10^6^/kg	32.69	2.84	209	7
(44)	SSc	2×10^6^/kg	1275	4.1	8262	9
(45)	SLE	1×10 ^8^	381	3.09	7478	16
(45)	SLE	1×10 ^8^	430	3.89	3376	9
(45)	IIM	1×10 ^8^	1488	5.05	12284	9
(45)	IIM	1×10 ^8^	2436	10.26	20153	9
(45)	SSc	1×10 ^8^	1260	4.07	8006	9
(46)	SSc	1×10^6^/kg	730	3.78	7130	10
(46)	SSc	1×10^6^/kg	47	–	1716	10
(46)	SSc	1×10^6^/kg	91	10.83	34439	10
(46)	SSc	1×10^6^/kg	1976	13.55	8737	10
(46)	SSc	1×10^6^/kg	163	9.58	2665	10
(46)	SSc	1×10^6^/kg	23	9.58	320	14
(46)	SSc	1×10^6^/kg	362	7.00	8553	10
(46)	SSc	1×10^6^/kg	117	5.57	1242	10
(46)	SSc	1×10^6^/kg	133	5.57	28020	7
(46)	IIM	1×10^6^/kg	10	12.44	61	10
(46)	IIM	1×10^6^/kg	465	5.35	14112	10
(46)	IIM	1×10^6^/kg	331	25.75	3020	10
(46)	IIM	1×10^6^/kg	324	5.29	10369	10
(46)	IIM	1×10^6^/kg	86	6.45	2579	7

Data were obtained from the summary of tables and figures in published literature, with graphical analysis performed using OriginPro and pharmacological analysis conducted using the DAS2 software platform. The table displays pharmacokinetic data from multiple independent studies utilizing different sample schedules, analytical methods, and CAR T-cell detection assays. Consequently, caution is warranted when directly comparing these parameters across studies. The primary goal of this compilation is to illustrate the scope and pattern of CAR T-cell proliferation in autoimmune conditions, rather than facilitating quantitative inter-study comparisons. Despite employing consistent digitization and non-compartmental modeling techniques to ensure internal coherence within each dataset, inherent discrepancies persist among the investigations.

We performed a systematic literature search using a predefined strategy to compile *in vivo* CAR T cell expansion data for this review. We searched clinicaltrials.gov, PubMed, and Web of Science. Search terms included “CAR T and Autoimmune disorders,” “CAR T and Autoimmune diseases,” “Anti-CD19 CAR T and Autoimmune diseases,” “Anti-CD19 CAR T and SLE,” and “Anti-CD19 CAR T and IIM”; the time window was limited to the most recent 6 years. Because our primary aim was to extract *in vivo* expansion metrics, we included only studies that directly reported relevant parameters (for example, Cmax and initial infusion dose) or provided clear, extractable images of CAR T cell expansion curves. We excluded studies that lacked key data, presented unclear expansion curves, showed error bars in the original figures, or reported only patient follow-up and prognosis. From clinicaltrials.gov we initially screened 36 clinical studies, of which 3 had published articles; of those 3, 2 did not report CAR T-cell expansion data, and the remaining article was excluded because the images were blurry, the patient expansion curves were indistinguishable, and the original authors did not provide raw data. From PubMed and Web of Science we retrieved 36 relevant articles. After review, we selected 20 articles that contained clear expansion curves or expansion data, yielding data on 42 patients for analysis. To avoid duplicate reporting, we cross-checked each downloaded article and categorized records by author, institution, and patient demographics to confirm no duplicate entries. Because preprints have not undergone peer review, their data’s authenticity and accuracy cannot be guaranteed; additionally, conference abstracts were screened but excluded from the quantitative dataset due to their general lack of detailed longitudinal data on CAR T-cell expansion, which is essential for pharmacokinetic modeling. Typically, these abstracts present only terminal outcome measures, such as response rates and complication incidences, without offering extractable expansion curves or serial cell-count data points needed for software-based pharmacokinetic/pharmacodynamic (PK/PD) analysis. Consequently, our quantitative PK/PD analysis dataset comprises only published research articles, case reports, and letters. However, recognizing the potential clinical significance of key conference abstracts that report major prospective trials, we discuss them separately as qualitative evidence in the subsequent sections. It is important to note that the data from these abstracts were not utilized in any quantitative modeling or software calculations. Therefore, only published research articles, case reports, and Letters were included. For pharmacokinetic parameters presented as images, we reproduced the data using the Image Digitization Tool in OriginPro. We imported the images and aligned the axes, performing multiple axis corrections for plots with discontinuous intervals to ensure each data point was reliable. We then captured points along the curves by manual selection or automatic tracking. Because the original authors had already marked the points and the plots lacked error bars, the digitization introduced minimal additional error; each curve was sampled three times, and the mean value was used to reduce random variation. The extracted data were entered into DAS software for pharmacokinetic analysis. Using non-compartmental modeling and the time–cell-count data, the software fitted the curves and computed T_max_, C_max_, and AUC. Because CAR T cells persist long-term *in vivo*, unlike conventional drugs, we report only AUC_0-t_ to ensure accuracy. The case data we have collected indicate that most patients achieved complete B-cell depletion within 5 to 10 days of receiving CAR T cell infusion, with this state persisting for 3 to 4 months. Although the duration of depletion may vary slightly among individual patients, it is complete within two weeks in all cases. At the same time, the majority of patients experienced immune reconstitution and recovery of immune function within a few months. This proves that anti-CD19 CAR T therapy can provide powerful remission in autoimmune diseases and supports dual regulation (24). From a pharmacokinetic perspective, however, CAR T cell expansion differs markedly between autoimmune diseases and studies. We found several explanations for this. First, autoimmune diseases differ in pathogenesis, so B cells localize and accumulate in different degrees. For example, in SLE B cells grow abundantly in blood and lymphoid compartments, while in RA B cells grow mostly in synovial tissue and produce autoantibodies (47). In MS, B cell proliferation is large in the central nervous system, creating heterogeneous niches for CAR T cell expansion that may explain variability in T cell proliferation observed in diseases (48). Study cohorts also differ in immune status, age, sex and prior treatments, and we show that comorbidities (e.g. diabetes) and advanced age can produce environments for CAR T cell expansion. Finally, variability in CAR T product designs (build design, manufacturing process, source) can influence post-infusion expansion. Differences in initial dose and treatment protocols also influence outcomes and contribute to heterogeneous growth of CAR T cell expansion.

Although anti-CD19 CAR T-cell therapy has proven effective in SLE, clinical evidence supporting its use in other autoimmune diseases remains limited. Notably, two recent large clinical studies—the CASTLE basket trial and the BREAKFREE-1 trial—provide important prospective data on the efficacy and pharmacodynamics of this therapy in systemic sclerosis (SSc) and idiopathic inflammatory myopathies (IIM).

The CASTLE trial was a Phase I/IIa basket study that enrolled 24 patients with refractory autoimmune diseases, including 10 with SLE, 9 with SSc, and 5 with IIM. All patients received a single infusion of the anti-CD19 CAR T-cell product zorpo-cel after discontinuing immunosuppressants and undergoing standard lymphodepletion (46). Pharmacodynamically, CAR T cells expanded in every patient, reaching a median peak concentration of 140 cells/μL. B cells were fully depleted within 7 days post-infusion, and the median duration of depletion was 83 days. Reconstituted B cells displayed a predominantly immature phenotype, consistent with an immune-reset mechanism. In efficacy assessments, none of the 9 SSc patients showed progression of interstitial lung disease at 24 weeks, and 4 of the 5 IIM patients met the ACR/EULAR major response; overall, 22 of the 24 patients met the pre-specified 6-month efficacy endpoints. In terms of safety, no patient experienced Grade ≥3 cytokine release syndrome (CRS) or any grade of immune effector cell-associated neurotoxicity syndrome (ICANS).

The BREAKFREE-1 trial assessed the efficacy of BMS-986353, a CD19 CAR T-cell product manufactured with the NEX-T platform, in 71 patients across three disease cohorts: SSc, SLE, and IIM. Pharmacologically, all cohorts exhibited robust CAR T-cell expansion, complete B-cell depletion, and reconstitution of immature B-cell phenotypes, consistent with findings from the CASTLE trial. In terms of efficacy, the ACR meeting abstract for the SSc cohort reported outcomes for 19 of 26 treated patients with available efficacy data. Among these, the six patients with baseline ILD demonstrated a median 10% increase in predicted forced vital capacity (pFVC) at 6 months versus baseline—an improvement not previously seen with other therapies (49). The ACR meeting abstract for the IIM cohort included 13 patients evaluable for safety and 11 for efficacy; of the evaluable patients, 91% achieved a moderate-to-severe composite improvement score, and muscle strength improved rapidly and substantially, with the median MMT-8 score rising by 22% at 6 months (50). Safety data from BREAKFREE-1 showed no severe CRS cases in the SSc and IIM cohorts but two Grade 3 ICANS events occurred—one in each of the SSc and IIM cohorts—and both patients made full recoveries.

In summary, data from the two independent trials, CASTLE and BREAKFREE-1, applied to autoimmune diseases other than SLE, show high consistency in pharmacodynamics, efficacy, and safety. Together, they demonstrate that anti-CD19 CAR T-cell therapy can induce deep B-cell depletion and an immune reset in SSc and IIM, yielding clinically meaningful control of disease activity and improvement in organ function. These prospective data provide stronger evidence to support the case-based efficacy reviewed here and offer a richer foundation for future quantitative pharmacodynamic modeling.

Based on the data we have collected so far, a robust quantitative pharmacology model is essential for the use of anti-CD19 CAR T-cell therapy in the treatment of autoimmune diseases. Such a model enables personalized treatment, allows for the prediction of therapeutic efficacy and risks, and provides guidance and support for CAR T-cell therapy in a highly heterogeneous patient population.

### 2.3 Practical challenges and risks in the clinical translation of anti-CD19 CAR T-cell therapy for autoimmune diseases

Although anti-CD19 CAR T-cell therapy holds substantial promise for treating autoimmune diseases, it faces significant practical challenges in cost, large-scale production, safety, and clinical translation (51).

The manufacturing workflow for autologous CAR T cells—the most commonly used form of anti-CD19 therapy—is highly individualized and involves sequential steps of cell collection, genetic modification, expansion, and reinfusion, which together yield very high overall treatment costs. These expenses limit access for many patients in the current resource-constrained healthcare environment (52, 53). In addition, because each patient’s T cells undergo separate processing, standardized batch production is difficult to achieve, which further increases the per-patient treatment cost (54).

In terms of production scaling, autologous therapies depend on a patient’s own T cells as the starting material for expansion. In some patients with autoimmune diseases, long-term immunosuppression or the underlying disease impairs T-cell number or function and thus compromises the success rate of CAR T-cell manufacture and the resulting product quality (54). Current efforts to overcome these limitations include automated closed-system bioreactors, non-viral gene-transfer methods (for example, mRNA or CRISPR editing), and the development of allogeneic “universal” CAR T cells; these approaches, however, remain exploratory and have not yet been implemented at scale for anti-CD19 CAR T therapy in autoimmune disease (54). Preliminary studies indicate that allogeneic anti-CD19 CAR T cells can function as an effective off-the-shelf treatment for systemic lupus erythematosus, offering a potential pathway to resolve production scalability challenges (55).

In terms of safety, cytokine release syndrome (CRS) is the most common and severe adverse reaction associated with anti-CD19 CAR T-cell therapy. CRS is triggered by the massive release of inflammatory cytokines (such as IL-6, IFN-γ, and TNF-α) following the activation of CAR T-cells, and can lead to clinical manifestations such as high fever, hypotension, dyspnea, and even multi-organ dysfunction (56). In patients with autoimmune diseases, although the disease background differs from that of malignant tumors, CRS still has a high incidence rate (33). In addition to common CRS, anti-CD19 CAR T-cell therapy in the treatment of autoimmune diseases also faces a unique local toxic reaction—local immune effector cell-associated toxicity syndrome (LICATS) (57, 58). LICATS is an organ-specific local reaction that occurs in patients with autoimmune diseases following CD19-targeted CAR T-cell therapy. Its mechanism may be related to the inflammatory response triggered by CAR T-cells during the clearance of pathogenic cells within local organs (59). An observational study showed that approximately 77% of patients experienced LICATS during treatment, manifesting as local inflammatory reactions in the affected organs (such as rash, joint swelling and pain, myalgia, and localized discomfort). Although the vast majority of cases were mild—with only a few reaching Grade 3 severity—and all patients experienced self-limiting resolution of symptoms without sequelae, LICATS, as a specific adverse event in the context of autoimmune disease, still requires thorough pre-treatment disclosure to patients and appropriate clinical management (59). Concurrently, off-target effects represent one of the primary risks associated with current CAR T-cell therapies. This refers to the situation where anti-CD19 CAR T-cells, while attacking pathogenic cells (such as autoreactive B cells), inadvertently damage normal tissues. In the treatment of autoimmune diseases, although CD19 is primarily expressed on B cells and theoretically exhibits strong specificity, there remains a potential risk of off-target damage to non-B-cell lineages under certain circumstances (60). Furthermore, anti-CD19 CAR T-cell therapy for autoimmune diseases aims to eliminate all B cells, including both pathogenic autoreactive B cells and normal protective B cells (37, 61). This results in persistent hypogammaglobulinemia in patients, significantly increasing the risk of infection. Although this falls under the “on-target” effect, in the context of autoimmune disease treatment, it is also regarded as an off-target consequence requiring long-term management. In response to the safety risks associated with anti-CD19 CAR T-cell therapy, some studies have attempted to enhance safety through novel technological approaches. For example, the development of chimeric autoantigen receptor T cells (CAAR T cells) designed to eliminate only pathogenic autoreactive B cells, as well as safety switch protocols capable of “emergency braking” to mitigate side effects such as CRS, provide new insights for creating safer, more effective, and smarter CAR T-cell therapies in the future (62, 63).

## 3 The role of quantitative pharmacology in CAR T therapy for autoimmune diseases

### 3.1 The value of quantitative pharmacology models in addressing clinical challenges

Quantitative pharmacology models characterize the complex *in vivo* mechanisms that govern disease and treatment, and they can generate virtual patient predictions by combining distinct mechanistic modules. By integrating mechanistic analysis, virtual simulation, and empirical data, these models transition CAR T therapy from an experience-driven practice to a prediction-driven paradigm. In the emerging application of autoimmune diseases, such models offer essential tools to address core clinical challenges, including uncertainties in cell kinetics, dose optimization, toxicity management, and patient-specific therapy selection. As regulatory agencies such as the FDA incorporate model-based analyses into the CAR T review process (64), model-guided precision development strategies will further advance the safe and effective use of CAR T therapies for autoimmune disorders.

To clarify the complex *in vivo* kinetics of live cells in CAR T–cell therapy, quantitative pharmacokinetic models simulate individualized expansion and clearance trajectories by integrating mechanistic modules such as CAR–antigen binding and T-cell phenotypic transitions (e.g., memory, effector, and exhausted states). In light of the complex dose–effect–toxicity relationships and pronounced interpatient heterogeneity, multidimensional models can generate virtual cohorts from real-world patient data. These virtual patients enable prospective clinical trial design and simulation of different dosing strategies—such as fractionated dosing and fixed-dose regimens—to evaluate effects on efficacy and to inform dose selection (65). Models can also track disease dynamics during treatment and predict their impact on subsequent CAR T efficacy, thereby aiding selection of optimal infusion timing, reducing failure from disease progression, and enabling “pre-treatment prediction” to guide regimen adjustments for high-risk patients (66). More broadly, quantitative pharmacology models reduce clinical risk and accelerate the development of novel CAR T therapies, offering distinct value to the field.

### 3.2 From tumors to autoimmune diseases: the need for a multi-scale quantitative systems pharmacology model

Most modeling of anti-CD19 CAR T–cell therapy in oncology has used semi-mechanistic approaches, with few comprehensive QSP models reported. Clinical translation of this therapy to autoimmune diseases faces unique and more complex challenges than in oncology, and these challenges provide the primary rationale for adopting comprehensive quantitative pharmacology models in autoimmune settings (67).

First, autoimmune diseases differ from tumors in the complexity of their pathogenic mechanisms and treatment objectives. Tumor therapies often rely on relatively simple elimination mechanisms, whereas autoimmune disorders involve multicellular interactions, organ-specific pathology, and dynamic perturbations of immune homeostasis that must be represented in models. Second, autoimmune diseases demand construction of comprehensive virtual patients because of profound patient heterogeneity, small population sizes, and complex tissue-specific pathophysiology (68). By contrast, hematologic malignancies typically display more homogeneous patient populations. Simplified, empirically based models built on average-patient data lack mechanistic depth and therefore cannot be reliably extrapolated to the highly heterogeneous autoimmune population (69). Finally, CAR T cell therapy for autoimmune diseases requires rigorous evaluation of safety and off-target risks (70, 71). Effective therapy must selectively eliminate pathogenic immune populations (for example, CD19^+^ B cells) without inducing excessive immunosuppression or collateral toxicity. Semi-mechanistic models that omit key pathophysiological mechanisms, microenvironmental interactions, and systemic feedback cannot adequately predict long-term outcomes of immune reconstitution.

A comprehensive QSP model addresses these challenges by integrating refined mechanistic modules to reveal microscopic mechanisms of action and to generate “virtual patients” for dynamic prediction (72). For example, coupling CAR T-cell kinetics with cytokine network modules and validating the model against real-world data enables effective prediction of cytokine release syndrome (CRS) risk. Thus, developing a comprehensive QSP model is essential for applying anti-CD19 CAR T-cell therapy to autoimmune diseases. It clarifies quantitative pharmacodynamic mechanisms, fills gaps in clinical data, and facilitates the clinical translation of this therapy toward personalized treatment.

## 4 Current status of quantitative mathematical modeling in the field of CAR T cell therapy

Quantitative models for CAR T cell therapy fall into two broad classes: semi-mechanistic cell kinetics–pharmacodynamics (CK-PD) or pharmacokinetics–pharmacodynamics (PK-PD) models, and quantitative systems pharmacology (QSP) models. CK-PD/PK-PD models use systems of ordinary differential equations to represent key processes such as CAR–antigen binding, T cell phenotypic conversion, expansion, and target-cell killing. These models capture clinical patterns of CAR T expansion, contraction, and persistence, and they can evaluate how dose, tumor burden, and antigen density influence exposure and efficacy (73). However, they typically omit multicellular interactions in the immune microenvironment, cytokine networks, and dynamic host-immune feedback; accordingly, they are best characterized as “semi-mechanistic” or “mechanistic but limited” and serve either as core modules within larger QSP frameworks or as simplified, tractable representations. By contrast, QSP models integrate multi-scale, multi-component interactions and incorporate clinically measurable early biomarkers as predictors. They can jointly analyze heterogeneous outcomes—complete remission, non-response, CD19^+^ relapse, and CD19^−^ relapse—and offer prospective predictive capability. QSP emphasizes comprehensive mechanistic coverage with clinical translation in mind, providing quantitative support for individualized treatment decisions rather than merely describing observed phenomena.

Below we summarize seven quantitative mathematical models currently used in CAR T-cell therapy, with a brief overview in **Table 2**. Five are semi-mechanistic oncology models, and two are full QSP models applied to oncology and to autoimmune disease, respectively. Although a few models address targets other than CD19 or include targets in addition to CD19, their core mechanisms and modeling frameworks align with those of anti-CD19 CAR T-cell therapy. Consequently, they offer useful insight and serve as references for developing idealized QSP models for autoimmune diseases; for this reason, we include them in our analysis.

**Table 2:** Summary of quantitative mathematical modeling in the field of CAR T-Cell therapy.

Reference	Type	True QSP	Target	Indications	Clinical endpoints
(74)	Semi-mechanistic PK-PD	NO	EGFR/HER2/CD19/BCMA	Various tumors (*in vitro*/mouse)	Tumor volume, CAR T concentration (Cmax/Tmax/AUC)
(76)	Semi-mechanistic CK-PD	NO	CD19	B-cell non-Hodgkin lymphoma	B-cell aplasia (surrogate for tumor burden), CAR T concentration
(75)	Semi-mechanistic PK-PD	NO	BCMA	Multiple myeloma	PFS, sBCMA, M-protein, response classification (CR/PR/SD/PD)
(77)	Population mechanistic	Approaching QSP	CD19	Large B-cell lymphoma	Tumor volume (estimated via SPD), IL-7/IL-15 correlation
(78)	Semi-mechanistic CK-PD	NO	CD19/CD22、GPRC5D/BCMA	B-ALL, multiple myeloma	CAR T expansion, tumor killing (*in vitro*/clinical)
(66)	QSP	YES	CD19	B-ALL	Response classification (CR/NR/CD19^+^/CD19^−^ relapse), relapse time prediction
(79)	QSP	YES	CD19	SLE	SLE-DAI score, biomarker dynamics, B-cell depletion and reconstitution

### 4.1 A review of model research in oncology

In terms of basic modeling, Singh et al. proposed a multiscale systemic PK–PD model in 2020 that exemplifies a semi-mechanistic PK–PD approach and establishes a quantitative link between *in vitro* efficacy parameters and *in vivo* outcomes. The model quantitatively relates CAR affinity, antigen abundance, tumor cell depletion, and CAR T cell expansion. Their work addresses the lack of quantitative tools to guide CAR design, screening, and *in vitro*–to–*in vivo* translation given that CAR T function depends on multiple factors such as CAR affinity, antigen density, and effector-to-target ratio. The model uses a three-step progressive strategy. First, a cell-level PD model was developed in which the number of CAR–target complexes drives target-cell killing, CAR T expansion, and cytokine release; saturation kinetics are described by a Hill equation. Second, a physiologically based pharmacokinetic (PBPK) model was constructed, partitioning organs (blood, lungs, liver, spleen, lymph nodes, and tumors) into vascular and extravascular compartments and assuming primary CAR T clearance via the liver. Finally, these components were integrated into a PBPK–PD model in which the number of CAR–target complexes concurrently drives tumor growth inhibition and CAR T expansion. Model outputs include target-cell killing rates, CAR T expansion multiples, IFN-γ release levels, *in vivo* tumor-volume dynamics, and blood CAR T concentration metrics (Cmax, Tmax, AUC). Validation used *in vitro* coculture data of anti-EGFR/HER2 CAR T cells with tumor cells of varying antigen densities and *in vivo* tumor-suppression and CAR T expansion data from anti-BCMA, HER2, CD19, and EGFR CAR Ts in xenograft mice. Key estimated parameters are that ~1.45 CAR–target complexes per tumor cell yield 50% of the maximum kill rate, and ~4.3 complexes yield 50% of the maximum expansion-promoting effect (74).

Building on the earlier framework, Singh et al. proposed a multiscale systemic PK–PD model one year later that used anti-BCMA CAR T (bb2121) as a case study. This model remains a semi-mechanistic PK–PD baseline model. Its introduction of a “threshold dose” offers direct guidance for clinical dose selection and can inform translational efforts from bench to bedside. The model seeks to clarify the poorly defined dose–exposure–response relationship of anti-BCMA CAR-T and to identify the pivotal drug-specific and system-specific parameters that shape clinical outcomes. Its structure extends the authors’ prior framework by partitioning the system into two compartments, blood and bone marrow.CAR T cells are divided into effector and memory phenotypes that are lost at different rates: effector cells have a short half-life, while memory cells have a long half-life. Effector cells convert to memory at a net first-order rate Rm. At the bone marrow site of action, CAR T cells form CAR–target complexes with tumor cells; the number of these complexes drives both effector CAR T cell expansion, characterized by a maximal expansion rate Kmax Exp and a half-maximal concentration EC50 Exp, and tumor cell killing, characterized by a maximal killing rate Kmax Kill and a half-maximal killing concentration KC50 CAR T.The model also includes two disease biomarkers—soluble BCMA and serum M protein—whose production rates are nonlinearly regulated by the tumor burden fraction. Outputs comprise the multiphasic CAR T blood kinetics (distribution, expansion, contraction, and maintenance), an indirect estimate of tumor burden, progression-free survival, and categorical responses: complete remission, partial remission, stable disease, and disease progression. Validation used *in vitro* killing data from six BCMA^+^ cell lines, results from a mouse RPMI-8226 xenograft model, and clinical PK, sBCMA, M-protein, and response data from 33 multiple myeloma patients (75). Virtual patient simulations revealed a CAR T dose “threshold effect”: increasing dose beyond approximately 150 million cells did not materially improve Cmax or response rates, whereas baseline tumor burden had a larger influence on exposure than CAR T dose.

In addition, Minucci et al. proposed a multiscale semi-mechanistic CK-PD model that characterizes CD4^+^ and CD8^+^ phenotypes in detail, thereby providing a framework to explain individual response variability and to link cell dynamics with pharmacodynamics in CAR T cell therapy. To address the clinical observation that CAR T cell expansion closely correlates with tumor burden—a pattern unexplained by conventional PK models—this study developed an integrated model that links molecular binding kinetics with cell-scale phenotypic differentiation. CAR T cells are represented as two lineages, CD8^+^ and CD4^+^, each subdivided into four phenotypes: naive, activated, effector, and memory. A system of ordinary differential equations governs phenotype transitions: naive cells activate at a defined rate; activated cells undergo a specified number of divisions with a characteristic division time before differentiating into effectors; effector cells convert to memory at a fixed fraction while the remainder die. The model explicitly represents CAR–CD19 binding and dissociation kinetics and uses the bound fraction as the proximate signal driving CD8^+^ effector–mediated B cell killing. Outputs comprise counts of CAR T cells by phenotype, B cell numbers as a surrogate for tumor burden, the CAR–antigen bound fraction, and the resultant killing rate. Model validation employed CAR T concentrations and B cell depletion data from 13 patients with non-Hodgkin lymphoma (76).

In terms of advanced modeling, Brown et al. developed a population-based mechanistic model to analyze the kinetic determinants of CAR T cells in lymphoma patients over a one-month period. The study sought to determine whether CAR T cell expansion in these patients is governed by both antigen-dependent and antigen-independent factors, although the precise mechanisms remain unresolved. The model uses a hierarchical, “Russian nesting doll” structure that expands from a baseline model containing only endogenous T cells to an integrated model that includes endogenous T cells, CAR T cells, four phenotypes (naive, central memory, effector memory, and terminal effector), and both blood and tumor compartments. A key innovation of the model is its separation of proliferation into antigen-independent and antigen-dependent components. Fitting blood flow cytometry and tumor biopsy data from 64 patients with relapsed/refractory non-Hodgkin lymphoma treated with Liso-Cel, the study found that endogenous T cell proliferation rates tracked serum IL-7 and IL-15 concentrations over time, with median Spearman correlation coefficients of 0.78 and 0.92, respectively. The model predicts population dynamics of endogenous T cells and CAR T cells in blood and tumors, the proportions of each phenotype, and tumor-volume changes estimated from tumor diameter. In virtual patient parameter-perturbation experiments, increasing the fraction of naive phenotype in the initial CAR T product by a factor of 10 had little effect on the total CAR T count during the first month, whereas increasing the endogenous proliferation rate by a factor of 1.5 markedly enhanced CAR T expansion. Sensitivity analysis identified tumor growth rate and antigen-dependent proliferation parameters as the primary drivers of tumor-volume changes, while phenotype-related parameters had little influence during the early phase (within 30 days) (77). This population-based mechanistic model aligns closely with the QSP framework and indicates that endogenous and CAR T cell expansion are tightly correlated; thus, monitoring endogenous T-cell dynamics in clinical settings may provide a surrogate marker for effective CAR T expansion.

In 2025, Su et al. proposed a quantitative pharmacology model for dual-targeted, dual-cistronic CAR T therapy based on multiscale mechanistic modeling. To address clinical challenges—unclear dose–exposure–response relationships and complex antigen escape mechanisms in bispecific CAR T cells—the study established the first quantitative framework linking *in vitro* and clinical settings. The *in vitro* component assumes bispecific CAR T cells coexpress anti-CD19 and anti-CD22 CARs and initially form monospecific complexes (CAR–CD19 or CAR–CD22). Some of these monospecific complexes then use the unbound CAR of the other specificity to form dual-target complexes (CAR–CD19–CD22). The model further assumes that all complexes exert equal, additive tumor-killing effects. Fitting data from co-cultures of CD19/CD22 CAR T cells with various tumor lines, the model estimated that approximately 3.57 CAR–target complexes per tumor cell are required to reach 50% of the maximal killing rate. When this *in vitro* killing model was integrated into the clinical CK–PD framework, the model further distinguished memory and effector CAR T populations, introduced two compartments (blood and bone marrow), and incorporated patient-specific CD19/CD22 antigen densities, the memory-to-effector ratio in the CAR T product, and age-adjusted body weight. The model accurately described qPCR and flow cytometry data from 14 B-ALL patients treated with the CD19/CD22 dual-target CAR T therapy AUTO3 and established a linear conversion between the two detection methods. Global sensitivity analysis showed that CD19 antigen density had a substantially greater effect on CAR T exposure than CD22 density, indicating that CD19 remains the dominant determinant in dual-target CAR T therapy. In addition, compared with single-target CAR T, the dual-target construct exhibited higher estimated maximal expansion rate (Kmax Exp) and maximal killing rate (Kmax Kill), consistent with the clinically observed superior complete remission rate (78). As a semi-mechanistic CK–PD model, this work provides the first quantitative pharmacology tool for dose optimization and construct screening of dual-target CAR T.

Among six tumor models, only one meets the true QSP standard framework. Liu et al. therefore proposed a computational immuno-oncology model to analyze and predict leukemia patient responses to CAR T cell therapy during remission, drug resistance, and relapse. The model integrates multiple mechanisms, predicts diverse clinical outcomes, and relies on clinically measurable variables. Its proposed early predictors can be translated directly into clinical decision-support tools and offer quantitative evidence to guide personalized treatment. Leukemia outcomes after CAR T therapy—including complete remission, non-response, CD19^+^ relapse, and CD19^−^ relapse—remain poorly understood and lack reliable early predictors. To address these challenges, they developed a quantitative systems pharmacology (QSP) model formulated as a system of ordinary differential equations. The model captures logistic growth of CD19^+^ B-ALL cells and their elimination by activated CAR T cells using Michaelis–Menten kinetics. It also represents CAR T cell activation, expansion in response to tumor cells, and apoptosis, together with the conversion and death of inactivated CAR T cells. To represent CD19^−^ relapse, we added a CD19^−^ tumor-cell compartment that accounts for mutational conversion from CD19^+^ to CD19^−^ cells and for bystander killing of CD19^−^ cells by CAR T cells. We then combined model variables into three composite indices: CAR T functional factors, negative relapse factors, and early predictive factors. After calibration and parameter estimation with clinical data from 209 B-ALL patients, the model reproduced observed CAR T and tumor-cell dynamics across response phenotypes (66). The model requires only the peak CAR T level and the AUC_7_ measured during the 7 days before treatment to predict long-term outcomes. When applied to a cohort of 1,600 virtual patients generated from real-world data, overall prediction accuracy reached 74.38%.

### 4.2 A review of research on autoimmune disease models

CAR T cell development for autoimmune diseases remains at an early stage, and researchers have not yet established many quantitative pharmacodynamic models to guide clinical practice. To date, only one quantitative pharmacology model for anti-CD19 chimeric antigen receptor T cells in systemic lupus erythematosus has been reported. Developed by Hyunseo Park’s team, this model systematically characterizes the dose–exposure–response relationship of CAR T cells in autoimmune environments and provides a theoretical basis for clinical dose optimization (79).

Identifying key pathophysiological processes is the initial step in model development. The model incorporates the following processes: CAR T cell dynamics that distinguish CD4+ and CD8+ subsets and capture their distinct kinetic behaviors across immune phenotypes; pharmacodynamics of lymphodepleting chemotherapy, modeling the effects of fludarabine and cyclophosphamide on host T-cell and neutrophil populations; CAR T targeting and killing of CD19+ B cells, represented by CAR–target complex formation kinetics; disease biomarker dynamics that link reactive B cell clearance to markers including anti-double-stranded DNA antibodies, complement C3, interferon-α, and proteinuria; and quantitative relationships between biomarkers and SLE disease activity indices derived by linear regression to enable prediction of clinical efficacy.

Second, the core components of the model architecture comprise two primary physiological compartments (peripheral blood and bone marrow) and include the following modules:

CAR T Cell Module: Describes the *in vivo* distribution, expansion, contraction, and persistence of infused CAR T cells. It separately tracks the differentiation and fate of the initial, central memory, effector memory, and terminal effector T cell subsets. Target Cell Depletion and Reconstitution Module: Simulates CAR T–mediated killing of self-reactive CD19+ B cells using CAR receptor–CD19 antigen binding kinetics. After reactive B cells are depleted and CAR T cell numbers fall below a defined threshold, the model initiates reconstitution of naive B cells originating from the bone marrow. The schematic diagram of this module is shown in **Figure 2**.

**Figure 2:**
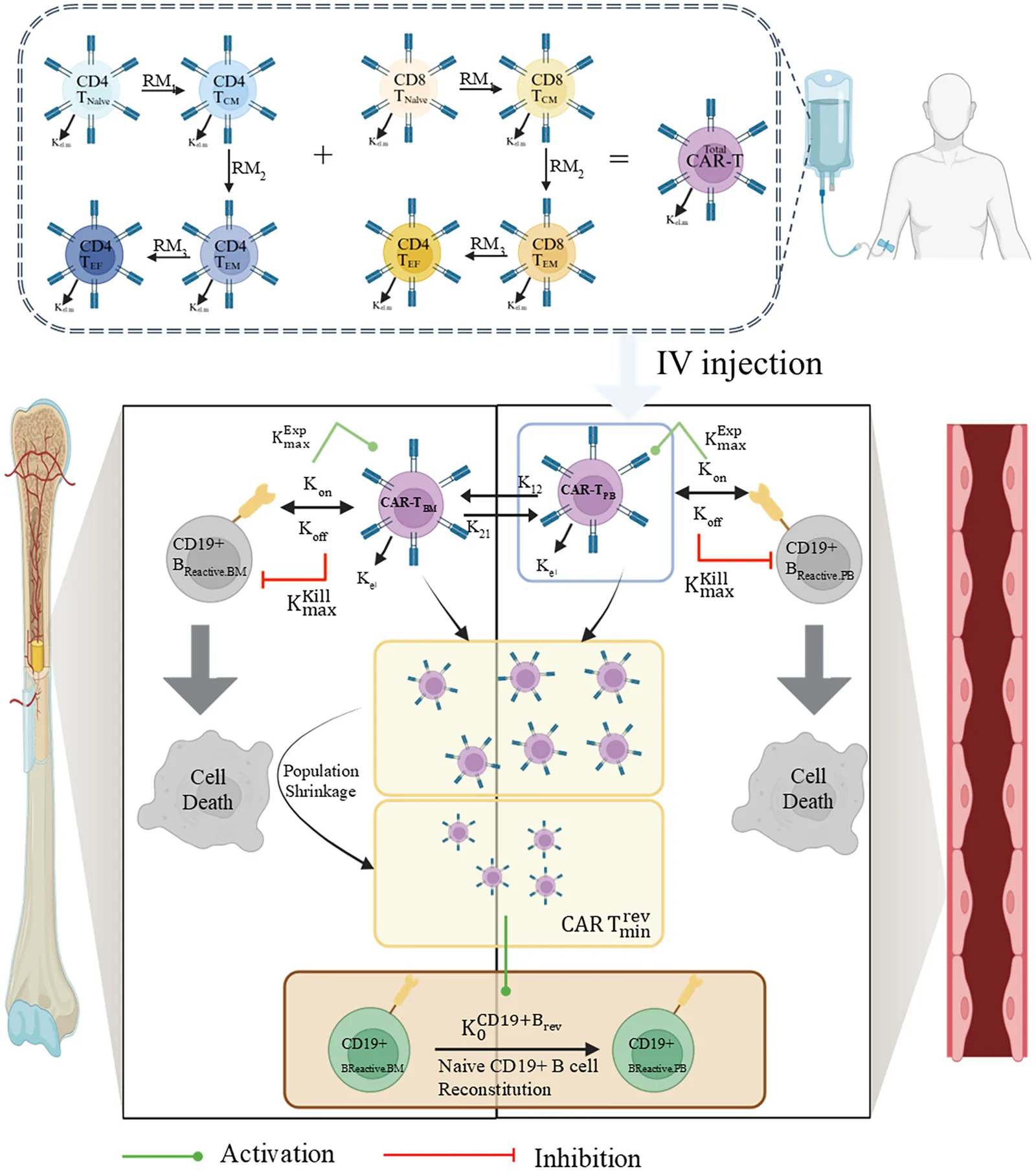
CAR T cell dynamics and CD19+ B cell depletion: This model assumes two physiological compartments: the systemic circulation and the bone marrow. Infused CAR T cells are divided into two subpopulations—CD4+ and CD8+—each comprising four major immunophenotypes: T_naive_ (naive T cells), T_CM_ (central memory T cells), T_EM_ (effector memory T cells), and T_EF_ (terminally differentiated effector T cells). Subsequently, the model assumes each subset distributes across compartments according to first-order distribution rates (K_12_ and K_21_). Differentiation from T_naive_ to T_EF_ follows first-order differentiation rate constants (RM1, RM2, and RM3, respectively). Within each compartment, CAR T cells interact with B cells, forming “CAR Target complexes” driven by K_on_ and K_off_, triggering killing of CD19+ B cells and CAR T cell population expansion. Once CAR T cells completely eliminate reactive B cells and the CAR T cell population contracts to a threshold (CAR Trev min), non-reactive naive B cells are assumed to be generated in the bone marrow at a zero-order rate constant (KCD19+Brev 0) and distributed to peripheral blood.

Lymphodepletion chemotherapy module: A pharmacodynamic model quantified the depletion of host CD4+, CD8+, and neutrophil populations following pre-treatment chemotherapy and characterized their subsequent recovery dynamics. The schematic diagram of this module is shown in **Figure 2**.

Disease biomarker module: A set of indirect-reaction models linked immune-cell kinetics to surrogate disease endpoints. The models posit that anti-dsDNA antibodies are produced by reactive B cells and are positively regulated by IFN-α (derived from plasma cell–like dendritic cells) and by CD4+ T cells. Reduced complement C3 levels and the onset of proteinuria are modeled as consequences of tissue damage and are correlated with neutrophil extracellular trap formation, CD8+ T cell infiltration, and IFN-α levels. The schematic diagram of this module is shown in **Figure 3**.

**Figure 3:**
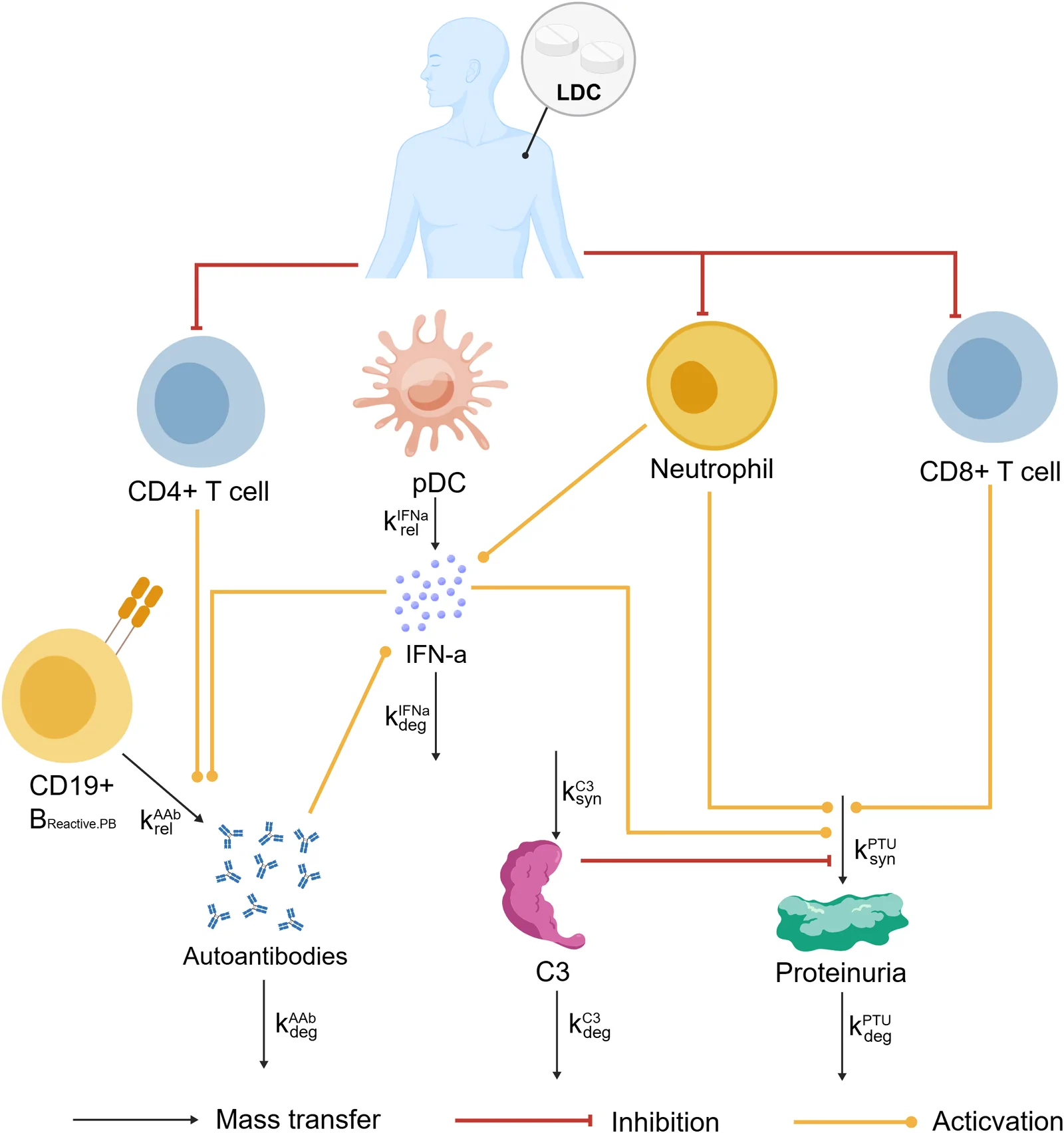
Relationship between host immune cells, reactive CD19+ B cells, and disease biomarkers: This model posits that the level of autoantibodies released by reactive CD19+ B cells represents the synthesis rate of anti-dsDNA autoantibodies, which are further activated by IFN-α secreted by CD4+ T cells and plasmacytoid dendritic cells (pDCs). CAR T cell elimination of reactive B cells attenuates and ultimately blocks this process, thereby reducing immune complex production. Proteinuria, a hallmark pathological feature of SLE, is thought to be activated by neutrophils via NETosis, CD8+ T cell-mediated immune responses, and the inflammatory cytokine IFN-α, but ultimately improves with reduced complement C3 (decreased formation of membrane attack complexes). The model hypothesizes that the abnormally elevated C3 protein in SLE patients decreases following clearance of anti-dsDNA autoantibodies. It accounts for all host immune cells involved in SLE pathophysiology, assuming they are affected after lymphocyte depletion chemotherapy (LDC) and that their numerical changes correlate with each cell type’s function. In the figure, KIFNa deg, KC3 deg, KPTU deg, and KAAb deg represent 1st order degradation rate constants of interferon alpha, C3, protein and anti- dsDNA auto-antibodies in peripheral blood respectively. KIFNa rel, KAAb rel represent 1st order release rate of interferon alpha from pDC and 1st order release rate of anti- dsDNA auto-antibodies from reactive B cells KC3 syn, KPTU syn represent 1st order synthesis rate of C3 in peripheral blood and Protein excretion in the urine per day.

Second, as a comprehensive QSP model, the logical chain that connects the clinical question, model outputs, and validation endpoints must be explicitly specified. The clinical question addressed by this model is the unclear dose–exposure–response relationship of anti-CD19 CAR-T cells in SLE patients, which has produced insufficient quantitative pharmacological guidance and hindered scientific optimization of dosing regimens. The model’s outputs derive from four interconnected submodules, with each submodule producing specific quantitative measures. In the lymphodepleting chemotherapy (LDC) module, the model simulates clearance and recovery kinetics of host immune cells—CD4+ T cells, CD8+ T cells, and neutrophils—after fludarabine and cyclophosphamide preconditioning, and it reports changes in host lymphocyte and neutrophil counts. In the CAR T cell dynamics (CK) and target-cell killing (PD) modules, the model describes distribution, expansion, contraction, and persistence of CAR T cells while distinguishing CD4/CD8 subtypes and immune phenotypes (Naive, CM, EM, and EF) after infusion. At the same time, it simulates recognition, binding, and elimination of CD19+ B cells by CAR T cells via immune-synapse formation, and it reports CAR T kinetic properties along with the depth of CD19+ B cell depletion and the time course of their recovery. In the disease biomarker dynamics module, the model employs multiple indirect-effect formulations to link upstream drivers—such as B-cell depletion and T-cell recovery—to synthesis and degradation of four key SLE biomarkers, and it produces their time-dependent trajectories. Finally, in the clinical-score (SLE-DAI) conversion module, the model uses linear regression to define an empirical relationship between those continuous biomarkers and the discrete SLE disease activity index, and it outputs the SLE-DAI score. After module construction, the researchers validated the main modules’ predictive reliability using internal and external long-term validation. For clinical outcomes, the QSP model’s primary predicted endpoint is a marked reduction in the SLE Disease Activity Index score, achieved via CAR-T–mediated depletion of CD19+ B cells and biomarker normalization, thereby producing clinical remission.

After defining the core clinical outcomes and validating the model data, the researchers fixed the validated model parameters and varied the input dose from the original 1×10^6^ cells/kg to 50%, 10%, 5%, and 1% to simulate exposure and response across doses. The model showed that 10% of the original dose (1×10^5^ cells/kg) is sufficient to eliminate reactive B cells and normalize biomarkers, thereby achieving the predicted clinical remission. This result directly addresses the clinical questions and informs personalized dosing strategies.

## 5 Construction of an idealized QSP model for autoimmune diseases with multiple diseases and multiple endpoints

The first quantitative systems pharmacology (QSP) model for systemic lupus erythematosus (SLE) represents a significant advance and offers a foundational architectural and methodological reference for applying QSP to autoimmune diseases. However, as an initial exploratory effort, it remains limited in its representation of pathological mechanisms and in its coverage of clinical phenotype complexity. In this review, we propose a framework to construct more comprehensive, idealized QSP models. We outline how to generalize a model developed for SLE to other autoimmune conditions and how to improve prediction of coupled, multi-endpoint clinical outcomes. Drawing on existing studies and available data, we present a practical conceptual approach to guide the development of next-generation QSP models.

### 5.1 Approaches and strategies for model construction

To achieve multi-disease coverage and multi-endpoint prediction and to address the clinical need for early, quantitative, and personalized prediction of efficacy, safety, and relapse risk in patients with autoimmune diseases treated with anti-CD19 CAR T-cell therapy, we propose a model framework composed of a “universal chassis + disease-specific plug-ins + tiered outputs.” The framework’s core design principle is “decoupling and reconfiguration,” which isolates the universal mechanisms of CAR T-cell therapy from the distinct pathophysiological mechanisms of individual autoimmune diseases and organizes them into a modular architecture of a universal chassis and specific plug-ins. It then produces hierarchical clinical endpoints relevant to personalized dosing, establishing a quantitative mapping from microscopic biomarkers to macroscopic clinical outcomes. Given the disease context and predefined endpoints, the framework automatically selects the appropriate parameter sets and output logic and thereby generates personalized dosing recommendations.

### 5.2 Model structure assumptions and key components

#### 5.2.1 General chassis section

This section describes the general mechanisms of CAR T cell therapy that form a core layer common to all diseases; the completeness of this layer determines the model’s capacity to represent autoimmune pathophysiology. We refined the prototype chassis from the SLE model by incorporating mature oncology models and additional complex mechanisms, producing a universal chassis composed of six major submodules. **Table 3** summarizes each submodule and highlights their novel features.

**Table 3:** The six major submodules of the universal chassis.

Submodule	Core function	Key innovation
Lymphodepletion Module	Simulates the depletion and recovery of host immune cells following Flu/Cy chemotherapy	Utilizes a K-PD model with precursor-dependent indirect response
CAR T Kinetics Module	CD4+/CD8+ dynamics, four-phenotype differentiation, distribution and expansion	Discrete division model, phenotypic differentiation chain, three-compartment system (blood – lymphoid organs – target tissues)
CAR Target Binding Module	Drives signal generation	Sequential binding of dual targets, extensible design
Target Cell Killing and Reconstitution Module	Differential dynamics of pathogenic vs. normal target cells	Forced distinction between two subpopulations, dual-source reconstitution
Cytokine Network Module	Three-axis feedback: pro-inflammatory, regulatory, and homeostatic	Mutual regulation of multiple cytokines, configurable
CAR T Exhaustion Module	Progressive loss of function	Exhaustion score-driven functional attenuation

##### 5.2.1.1 Lymphocyte depletion module

Lymphodepletion chemotherapy (LDC) aims to reduce host lymphocytes to create a niche for anti-CD19 CAR T-cell expansion and to trigger release of homeostatic cytokines (IL-7, IL-15) (80, 81). A mature lymphocyte chemotherapy module has been validated in SLE patients and reliably captures lymphopenia and subsequent recovery after LDC. Because clinical autoimmune datasets often lack drug concentration measurements, we implemented a K-PD (kinetic–pharmacodynamic) model—validated in SLE—that uses virtual drug concentrations to drive the pharmacodynamic effects.

##### 5.2.1.2 CAR T-cell kinetics module

In this module, we distinguish CD8+ and CD4+ CAR T cells, each subdivided into four phenotypes: naive, central memory (CM), effector memory (EM), and effector (EF). We adopt the discrete-division model proposed by Minucci et al. to describe T-cell activation and division. In this model, each activated cell divides ndiv times during a differentiation period τ, and a fraction of the progeny ultimately acquires a memory phenotype.

For phenotypic differentiation, we adopted the first-order irreversible rate constants from the SLE model and set the rates for T-cell progression from Tnaive to TCM, TEM, and TEF as RM1, RM2, and RM3, respectively. For cell distribution, we replaced the SLE model’s two-compartment circulation–bone marrow scheme with a three-compartment architecture comprising peripheral blood, lymphoid organs, and inflammatory target organs. The inflammatory target–organ compartment is implemented as a disease-specific plugin, permitting CAR T–cell distribution to vary by autoimmune disease. For expansion dynamics, we implemented a Cplx-triggered proliferation mechanism based on the Hill equation from Singh et al., 2020. Finally, we formulated a discrete division model to represent CAR T–cell activation and division, thereby aligning the model more closely with the biological reality of T-cell activation (82).

##### 5.2.1.3 CAR-target binding module

Within the target-binding module, CAR binding to the antigen follows the law of mass action and forms a CAR–target complex (Cplx). The Cplx serves as a unified signaling driver that concurrently governs target-cell killing, CAR T-cell expansion, and cytokine release. Although this study focuses on CD19 CAR T-cell therapy, motivated by the current need for multi-target strategies and by the work of Su et al., the module can adopt a sequential-binding hypothesis for dual-target CARs (e.g., CD19/CD22): a single-target complex forms first, and a fraction of those complexes subsequently converts into a dual-target complex.

##### 5.2.1.4 Target cell killing and reconstruction module

In this module, we differentiate pathogenic and normal cell subpopulations, a key feature of autoimmune diseases. The pathogenic subpopulation produces autoantibodies and cytokines at elevated rates, whereas the normal subpopulation retains baseline turnover and immune function. Proliferation rates, antigen density, sensitivity to CAR T–cell cytotoxicity, and recruitment sources are specified independently for each subpopulation. We extend the SLE model’s “reactive B cells + naive B cells” formulation to a generalized “pathogenic + normal” dual-subpopulation framework. We also replace the single recruitment source with a dual-source scheme—neogenesis plus memory-cell expansion—to better capture heterogeneous recruitment patterns observed in autoimmune disorders.

##### 5.2.1.5 Cytokine network module

Unlike the single-factor IFN-α axis used in the SLE model, we expanded this module to a three-axis cytokine network comprising a pro-inflammatory axis, a regulatory axis, and a homeostatic axis. Cytokine production rates are proportional to the abundances of specific cell types (pathogenic B cells, pDCs, macrophages, CAR T cells, etc.), and clearance follows first-order kinetics. The detailed structure of the three-axis network is presented in **Table 4**. We also include mutual-regulation equations to represent positive and negative feedbacks among cytokines—for example, IFN-α enhancing IL-10 production and IL-10 suppressing CAR T cell expansion—thereby capturing the complex feedback mechanisms characteristic of autoimmune disease. For each disease plugin, the system activates only the 2–3 most relevant cytokines and either deactivates or fixes the remaining ones to avoid overparameterization.

**Table 4:** Summary of cytokine tri-axis network regulation.

Cytokine axis	Key cytokines	Major cellular sources	Primary functions	References
Pro-inflammatory Axis	IFN-α, TNF-α, IL-6, IL-17, GM-CSF	pDCs, Macrophages, Th17 cells	Amplification of inflammation, tissue damage	(111, 112)
Regulatory Axis	IL-10, TGF-β, BAFF	Tregs, Bregs, Macrophages	Immunosuppression, B cell survival	(113, 114)
Homeostatic Axis	IL-7, IL-15	Stromal cells, DCs	T cell survival and basal proliferation	(115–117)

##### 5.2.1.6 CAR T-cell exhaustion module

In autoimmune diseases, CAR T cells experience persistent, low-level antigen stimulation that drives an exhaustion pattern similar to the progressive functional decline seen in chronic infections (83). Here, we define an exhaustion score (E_Score) that depends on cumulative Cplx exposure, and we model CAR T cell expansion, cytotoxicity, and memory conversion efficiencies as decreasing functions of E_Score. This formulation reflects the biological reality of gradual T cell functional loss. We also enable configurable exhaustion accumulation rates within the disease module to capture how different inflammatory environments can produce varying CAR T cell exhaustion kinetics across contexts (84).

#### 5.2.2 Disease plugins section

Based on prevalence, the strength of clinical evidence, and the uniqueness of pathological mechanisms, we selected five autoimmune disease categories as the initial targets for disease module implementation: systemic lupus erythematosus (SLE), systemic sclerosis (SSc), idiopathic inflammatory myopathies (IIM), rheumatoid arthritis (RA), and multiple sclerosis (MS). Each disease plugin follows a “generic chassis + disease-specific extensions” design. Using the six submodules of the generic chassis, different autoimmune disorders are accommodated by replacing or refining four core components: target cell definitions (cell types, markers, and kinetics of pathogenic and normal subpopulations); cytokine networks (activated nodes and regulatory interactions); tissue damage modules (target organs, damage markers, and kinetic models); and clinical score mapping (quantitative relationships between biomarkers and clinical scores). The SLE model is well characterized and can be applied directly; for the remaining diseases, we next describe the key mechanisms and the composition of each plugin, and we specify the required data sources in the following section.

##### 5.2.2.1 Systemic sclerosis plug-in

The central pathophysiological framework of SSc is the “vascular pathology–immune activation–fibrosis” triad. B cells drive fibroblast activation and collagen deposition through production of autoantibodies, such as anti-topoisomerase I (anti-Scl-70), and by secreting cytokines, notably IL-6 (85, 86).

In the target cell differentiation section, since the primary target cells for CD19 CAR T cells in this disease are autoreactive B cells and plasma cells, target cells were divided into pathogenic subgroups (autoreactive B cells/plasma cells) and normal subgroups (naive B cells, Bregs). In the cytokine network section, we selected TGF-β, IL-6, and IL-13 from the activation common chassis to construct a cytokine network for SSc (87, 88). In the tissue damage section, we incorporated skin scoring (mRSS), pulmonary function indicators (FVC%, DLCO%), and anti-Scl-70 antibodies as markers of damage. Finally, in the clinical score mapping section, we employed the ACR CRISS (American College of Rheumatology Scleroderma Composite Response Index) to perform projections by integrating changes in mRSS, FVC%, and physician/patient global assessments (89, 90).

##### 5.2.2.2 Idiopathic inflammatory myopathies plug-in

IIM is marked by muscle inflammation driven by B cells, CD4+ T cells, dendritic cells, and macrophages. B cells generate myositis-specific antibodies (MSAs, such as anti-Jo-1, anti-MDA5, and anti-TIF1-γ) (91), and the type I interferon pathway is central to dermatomyositis (92, 93).

In the target cell differentiation section, mirroring the approach used for SSc, we distinguish pathogenic subpopulations (autoreactive B cells/plasma cells) from normal subpopulations. In the cytokine network section, we include activation of type I IFN (IFN-α/β) and IL-6 within a universal activation chassis as core components of the disease cytokine network (94). For the tissue damage section, we use muscle strength (MMT-8), creatine kinase (CK), and MSA titers as damage markers (95). In the clinical score mapping section, we apply the ACR/EULAR Myositis Response Criteria, integrating MMT-8, CK, physician and patient global assessments, and the Health Assessment Questionnaire (HAQ) as clinical score projections (96, 97).

##### 5.2.2.3 Rheumatoid arthritis plug-in

Rheumatoid arthritis (RA) is driven primarily by aberrant activation of synovial fibroblasts (FLS), which produce invasive synovial tissue that destroys cartilage and bone. B cells also contribute by secreting rheumatoid factor (RF) and anti-CCP antibodies (98). This plugin addresses RA pathogenesis and supports two complementary CAR T strategies: a B-cell pathway that targets CD19^+^ B cells and plasma cells, and an FLS pathway that targets activated FLS expressing ITGA5, CD142, or FAP.

In the target cell differentiation section, we adopt a dual-pathway framework: the B-cell pathway distinguishes pathogenic autoreactive B cells and plasma cells from normal B-cell subpopulations, while the FLS pathway specifies pathogenic activated FLS subpopulations characterized by ITGA5^+^, CD142^+^, and FAP^+^ expression. In the cytokine network section, we selected TNF-α, IL-6, and GM-CSF from the common activation chassis as key cytokines for RA (99, 100). For tissue-damage markers, we included swollen and tender joint counts (SJC28/TJC28), the Sharp score (radiographic erosions), anti-CCP antibodies, and CRP (101). Finally, we mapped clinical outcomes using DAS28-CRP as the clinical score.

##### 5.2.2.4 Multiple sclerosis plug-in

The traditional model of MS attributes pathology to CD4+ T cells (Th1/Th17) that attack the central nervous system myelin sheath; however, the clinical efficacy of anti-CD20 monoclonal antibodies (ocrelizumab, rituximab) indicates a critical role for B cells in disease initiation (102, 103). B-cell follicle-like aggregates in the meninges have also been closely associated with clinical progression.

In the target cell classification, we divided cells into pathogenic subgroups (autoreactive B cells and plasma cells) and nonpathogenic subgroups. In the cytokine network, we identified IL-17, IFN-γ, and GM-CSF in the common activation pathway as key cytokine components (104–106). For tissue damage, we used the number of new MRI lesions, brain atrophy volume, and the cerebrospinal fluid oligoclonal band/IgG ratio as representative measures. Finally, in clinical score mapping, we implemented the Expanded Disability Status Scale (EDSS) and the Multiple Sclerosis Severity Score (MSSS) (107, 108).

#### 5.2.3 Clinical endpoints, outcomes, and decision-making section

In the second-layer disease plug-in, we specify disease-specific target cells, cytokines, tissue-damage markers, and equations that map those biomarkers to clinical scores. The third layer applies these mapping equations to translate the biomarker outputs from the second layer into clinical scores. Risk thresholds and efficacy criteria used by the third layer—such as CRS grading, B-cell depletion depth, and remission definitions—are derived from the clinical trial data employed in the second layer, enabling a seamless transition from mechanism-based simulation to clinical outcomes.

For safety endpoints, we developed a system that estimates the probability of CRS and ICANS using simulated indicators—such as cytokine levels (IL-6, IFN-γ) and lymphocyte counts—generated by the first and second layers, thereby informing decisions on prophylactic medication and dose adjustment. For pharmacodynamic endpoints, we implemented a model that quantifies biological response to CAR-T therapy by simulating the extent of pathogenic target-cell depletion and the time to cell repopulation from the first and second layers, thus guiding decisions on second infusions or combination therapies. For clinical benefit endpoints, we created an evaluative framework that predicts overall patient outcomes and long-term organ protection using simulated clinical scores and steroid dosages derived from the first and second layers, to inform maintenance regimens and the timing of treatment discontinuation.

Finally, to enable direct clinical use, we developed a system that combines virtual patient simulation with the Classification and Regression Trees (CART) algorithm. The system accepts patient baseline characteristics and model predictions as inputs and outputs specific treatment regimens—dosage, infusion method, combination therapies, and monitoring plans—to guide clinical dosing decisions based on the model.

### 5.3 List of model parameters and data requirements

The mathematical structure of the submodules in the general chassis of Part I builds on published CAR T models for cancer and autoimmune diseases. Its main innovation is the integration of disparate modules into a unified platform and the adaptive expansion of parameter ranges to reflect autoimmune-specific features such as low antigen load, chronic inflammation, and the need for immune reconstitution. In this section, we therefore describe the key parameters of each module and the rationale for their selection, summarized in **Table 5**.

**Table 5:** Parameters of the universal chassis and their sources.

Module	Parameter	Description	Value/distribution	Source
LDC	K_LD_	Virtual elimination rate of LDC (1/day)	1.39 (RSE 10.8%)	(79)
LDC	${\text{E}}_{\max}^{\text{cd}4}$	Maximum inhibition of CD4+ T cells by LDC	0.85	(79)
LDC	${\text{E}}_{\max}^{\text{cd}8}$	Maximum inhibition of CD8+ T cells by LDC	0.96	(79)
CAR T	${\text{E}}_{\exp}^{\max}$	Maximum expansion rate (1/day)	2.04 (RSE 5.21%)	(78)
CAR T	${\text{E}}_{\exp}^{50}$	Half-maximal effective complex number	42.1 (RSE 34.0%)	(78)
CAR T	${\text{K}}_{e}^{\text{el}}$	Elimination rate of effector cells (1/day)	113 (fixed)	(78)
CAR T	${\text{K}}_{m}^{\text{el}}$	Elimination rate of memory cells (1/day)	0.015 (RSE 31.7%)	(78)
CAR T	K_12_	Blood-to-tissue distribution rate (1/day)	2.91 (RSE 47.2%)	(78)
CAR T	K_21_	Tissue-to-blood redistribution rate (1/day)	0.18 (fixed)	(78)
Binding	K_on_	Association rate constant (1/M·s)	1×10⁵ (fixed)	(74)
Binding	K_off_ (CD19)	Dissociation rate constant (1/s)	1.09×10⁻⁴	(118)
Binding	Density CAR	CAR density (molecules/cell)	15,000 (fixed)	(78)
Killing	${\text{K}}_{\text{kill}}^{\max}$	Maximum killing rate (1/day)	0.343 (RSE 21%)	(75)
Killing	${\text{KC}}_{\text{kill}}^{50}$	Half-maximal effective complex number	2.24 (fixed)	(74)
Killing	τ_kill_	Killing delay (days)	7.84 (RSE 36.7%)	(75)
Exhaust	K_exhaust_	Accumulation rate of exhaustion (1/day)	To be calibrated	—
Exhaust	K_recover_	Recovery rate from exhaustion (1/day)	To be calibrated (<0.001)	—

Regarding the disease modules in Part II, except for SLE, clinical data on CD19 CAR-T therapy for other autoimmune diseases remain scarce. This section therefore enumerates the complete set of data required to calibrate each disease module and systematically compiles published clinical trial data and alternative data sources to support subsequent model calibration and external validation.

First, model calibration requires five categories of standardized data regardless of disease modality (1): patient baseline characteristics, including age, sex, weight, duration of illness, history of prior B-cell depletion therapy and response, and baseline disease activity score (2); CAR T product characteristics, including infusion dose, CD4/CD8 ratio, and the percentage of each phenotype (Naive/CM/EM/Effector) (3); CAR T-cell kinetics, including the concentration–time curve of CAR T cells in peripheral blood (which we have attempted to describe in **Table 1**) (4); target cell kinetics, including changes in absolute B-cell counts and the proportions of various subsets; and (5) disease biomarkers, including autoantibody titers, cytokine concentrations, and indicators of tissue damage. In addition, safety data—such as CRS grading, ICANS, infections, and other adverse events—and clinical outcomes, including disease activity scores, remission rates, and time to relapse or progression, must be collected.

For diseases other than SLE, we compiled recent clinical data as follows:

Systemic sclerosis (SSc): The primary data sources include the CASTLE basket trial (see Section 2.2 for details) and the BREAKFREE-1 trial, as well as the SSc cases reported by Müller et al. in **Table 1**.

Idiopathic inflammatory myopathies (IIM): The primary data sources include the CASTLE basket trial and the BREAKFREE-1 trial, as well as the cases reported by Pecher et al. and Nicolai et al. in **Table 1**.

Rheumatoid arthritis (RA): Direct clinical data on CD19 CAR T therapy for RA remain limited. We identified a single case report in which a patient with long-standing RA achieved sustained, drug-free remission after treatment with a CD20–CD19 dual-targeted CAR T cell therapy (zamtocabtagene autoleucel). This is the first published clinical case of CAR T therapy for persistent, long-standing RA (109).

Multiple sclerosis (MS): Clinical data supporting CD19 CAR T-cell therapy in MS are limited but not entirely absent. In our case series (**Table 1**), two MS patients underwent anti-CD19 CAR T-cell treatment, with detailed outcomes documented in reference 36. Although these cases offer initial feasibility evidence, the sample size is insufficient for generalizable conclusions. Apart from these cases, indirect and preliminary evidence is arising from alternative CAR T-cell approaches. For example, Qin et al. published a Phase I study in Cell on anti-BCMA CAR T therapy in five progressive MS patients. (1 with primary progressive MS and 4 with secondary progressive MS) (NCT04561557). Treatment depleted plasma cells in the central nervous system, and CAR T cells showed sustained expansion with reduced depletion in cerebrospinal fluid. Overall safety was acceptable, providing an important proof of concept for CAR T–based approaches in MS (110). Several early-stage CD19 CAR T trials are now being registered and initiated for MS, and additional data are expected in the future.

For the third layer—clinical outcome and decision-making—the data and parameters to be collected fall into three primary categories. Unlike the inputs for the first and second layers, this layer emphasizes clinical outcome data tied to decision thresholds rather than mechanistic parameters. Because such data are currently scarce in clinical settings, we provide only a brief overview here and summarize the findings in **Table 6**, with the expectation that future research will augment the data for this module.

**Table 6:** Clinical endpoint outputs and core data required by decision-makers.

Category	Specific data/parameters	Purpose
Safety Endpoints	CRS grade (1–4), ICANS grade, peak concentrations of IL-6/IL-2/IFN-γ, lymphocyte count	To establish risk prediction models for CRS/ICANS and to set preventive intervention thresholds
Pharmacodynamic Endpoints	Depth of B-cell depletion, duration of depletion, B-cell reconstitution time, reduction rate of autoantibodies	To evaluate biological response and to determine the need for reinfusion or combination therapy
Clinical Benefit Endpoints	Disease activity scores, rate of glucocorticoid discontinuation, DORIS remission, ACR CRISS response, ACR/EULAR response, progression-free survival (PFS), cumulative damage indices	To ascertain long-term efficacy, maintenance regimens, and timing of treatment withdrawal
Data Required for Decision-Tree Training	Paired data of patient baseline characteristics, model-predicted outputs, and actual clinical outcomes	To train CART decision trees and generate personalized dosing rules

### 5.4 Challenges and limitations in building idealized models

The proposed “disease-endpoint” two-dimensional response matrix QSP model is grounded in theory and supported by initial research on anti-CD19 CAR T-cell therapy for autoimmune diseases; it could shift quantitative pharmacology from a descriptive discipline to a decision-making tool. However, under current data and technical constraints, full implementation is not feasible. As a novel indication for CAR T-cell therapy, clinical data on autoimmune diseases are extremely limited. The multi-disease, multi-endpoint, high-quality longitudinal datasets required to build the model do not exist, and the parameter complexity would exceed what can be reliably calibrated with available data. Accordingly, this review outlines only an idealized blueprint for a future model. A more pragmatic strategy is to begin with the extant SLE model and expand iteratively in two directions—horizontal multi-disease and longitudinal multi-endpoint—with each expansion validated rigorously against extensive data. As clinical experience accumulates, a practical QSP model is expected to be developed. The model will initially focus on three diseases—systemic lupus erythematosus (SLE), systemic sclerosis (SSc), and idiopathic inflammatory myopathies (IIM)—for which abundant clinical data and established efficacy exist. Because direct evidence is currently limited, the model will not yet cover rheumatoid arthritis (RA) or multiple sclerosis (MS), although it may be extended to them as research advances. The model will generate six key endpoints: CRS/ICANS risk probability, depth of pathogenic target cell depletion, B-cell recovery time, improvement in clinical scores, corticosteroid dose and timing of discontinuation, and relapse-free survival. These endpoints draw on real-world data and basket trial results summarized in Section 2.2, integrating safety, pharmacodynamics, and clinical benefit to inform personalized treatment strategies.

## 6 Summary and outlook

This review examines the therapeutic potential of anti-CD19 CAR T-cell therapy for autoimmune diseases, with an emphasis on the role of quantitative pharmacology models in optimizing dosing, safety, and efficacy, and on the current state of clinical research. Anti-CD19 CAR T-cell therapy presents transformative promise for autoimmune disorders, but its clinical success requires precise risk management and individualized dosing (46). Therefore, quantitative pharmacology models serve not only as tools for characterizing the complex *in vivo* behavior of CAR T cells, but also as the scientific basis for developing rational, patient-specific dosing strategies and predicting risks and therapeutic outcomes. Advancing quantitative pharmacology and integrating it with clinical practice, basic immunology, and novel engineering approaches will help address current challenges in CAR T-cell therapy for autoimmune diseases and support the development of safe, effective, and durable regimens tailored to each patient.
